# Quantification of HBsAg to predict low levels and seroclearance in HBeAg-negative patients receiving nucleos(t)ide analogues

**DOI:** 10.1371/journal.pone.0188303

**Published:** 2017-11-30

**Authors:** Teresa Broquetas, Montserrat Garcia-Retortillo, Juan José Hernandez, Marc Puigvehí, Nuria Cañete, Susana Coll, Beatriz Cabrero, Maria Dolors Giménez, Ricard Solà, José A. Carrión

**Affiliations:** 1 Liver Section, Gastroenterology Department, Hospital del Mar, Universitat Autònoma de Barcelona, Departament de Medicina, Barcelona, Spain; 2 IMIM (Hospital del Mar Medical Research Institute), Barcelona, Spain; 3 Laboratori de Referencia de Catalunya (LRC), El Prat de Llobregat, Barcelona, Spain; Centre de Recherche en Cancerologie de Lyon, FRANCE

## Abstract

**Background:**

HBeAg-negative chronic hepatitis B patients require long-term nucleos(t)ide analogues(NAs) because loss of surface antigen (HBsAg) is unusual. Low quantitative HBsAg (qHBsAg) levels can identify patients with higher probability of seroclearance. The aim of our study was to evaluate qHBsAg in HBeAg-negative patients receiving NAs to predict a reduction of HBsAg levels and seroclearance.

**Methods:**

Retrospective analysis of qHBsAg in HBeAg-negative patients before and at years 1, 3, 5, 8 and over of NAs treatment.

**Results:**

From 1999 to 2015, HBsAg was quantified in 358 serum samples from 95 HBeAg-negative patients. Low qHBsAg (<120 IU/mL) was identified at baseline or during follow-up in 14% of patients and HBsAg loss in 4%. No baseline variables predicted seroclearance and only treatment duration predicted low qHBsAg. The annual decline of qHBsAg was -0.102 log IU/mL and the median time to HBsAg loss was 6.04 years. The decline was greater in patients achieving low HBsAg levels (-0.257) than in those who did not (-0.057)(p<0.001). The diagnostic accuracy (ROC curve, 95%CI) of qHBsAg delta at year 3 was 0.89 (0.81–0.97), with cut-off >0.3 log IU/mL showing a positive and negative predictive value of 42% and 100% to identify patients achieving low levels of HBsAg.

**Conclusions:**

Reduction of qHBsAg is slow in HBeAg-negative patients receiving NAs, although low levels or faster qHBsAg decline may occur in 14%. A qHBsAg reduction >0.3 log IU/mL at year 3 can identify patients with a higher probability of achieving low levels and HBsAg seroclearance.

## Introduction

The therapeutic endpoint in chronic hepatitis B (CHB) is the functional cure defined as sustained off-drug suppression of serum hepatitis B surface antigen (HBsAg), hepatitis B virus (HBV)-DNA and covalently close circular (ccc)DNA[1]. However, this endpoint is rarely achieved with the available therapies in HBeAg-negative. Serum HBsAg is the glycosylated envelope protein of the mature HBV which is produced by transcription and translation of the surface genes[2]. It has been suggested that serum HBsAg could be considered as a surrogate marker of cccDNA and a marker of host immune control of HBV infection[3]. HBsAg levels significantly vary during HBV infection showing a reduction from HBeAg-positive to HBeAg-negative infection[4].

HBsAg quantification (qHBsAg) has recently been proposed as an important tool in clinical practice to identify HBeAg-negative infected patients (former inactive carriers). Brunetto et al. have shown that the combination of qHBsAg <1000 IU/mL and HBV-DNA ≤2000 IU/mL can recognize most inactive carriers[5]. Another use of qHBsAg may be monitoring antiviral response in HBeAg-positive or -negative patients receiving pegylated interferon (Peg-IFN)[6, 7]. Peg-IFN has immune-mediated antiviral activity which modulates HBsAg production and secretion[8]. Moreover, it has been proposed that qHBsAg during nucleos(t)ide analogues(NAs) therapy can provide additional information to HBV-DNA levels. However, the decline of qHBsAg during NAs therapy is less pronounced than that observed in patients receiving Peg-IFN[8, 9]. A rational explanation is that NAs block viral reverse transcriptase, inhibiting HBV-DNA, but this does not affect either cccDNA or HBsAg production[10].

The qHBsAg has been proposed as a predictor of HBsAg clearance in patients receiving NAs[11–13]. Seto et al. have shown that 10% of patients receiving LAM achieved HBsAg-clearance[12] during the follow-up. Moreover, the decline of qHBsAg was similar in HBeAg-positive and -negative patients, being around -0.104 log IU/mL/year. These authors identified that HBsAg-clearance occurred in patients with a low baseline qHBsAg (<1000 IU/mL) and high on-treatment reduction (>0.166 log IU/mL per year) with a negative predictive value (NPV) of 98%. The BE-LOW study demonstrated similar results using entecavir (ETV)[14]. However, the reduction in qHBsAg was greater in HBeAg-positive than in -negative patients. Recently, a large European cohort receiving tenofovir (TDF) showed that a reduction of qHBsAg levels <1log at weeks 12 and 24 had a NPV to identify HBsAg-loss of 94% and 97%, respectively[15]. Additionally, Chen et al. have demonstrated that qHBsAg could be useful in finite therapies with NAs, to select patients with low risk of relapse after discontinuation. A qHBsAg level<120 IU/mL at the end of treatment (EOT) could predict 79.2% of HBsAg loss in HBeAg-negative patients in whom LAM was discontinued[13].

In contrast, other studies have concluded that NAs produce a very slow decline of qHBsAg and several years are necessary to identify HBsAg-clearance [12, 16]. In a recent Spanish prospective registry, the HBsAg loss rate in HBeAg-negative patients under tenofovir (TDF) or entecavir (ETV) treatment was only 0.3% after five years of follow-up[17]. Therefore, the role of qHBsAg in patients receiving NAs remains unclear with limited data in HBeAg-negative patients.

Thus, the aim of our study was to evaluate qHBsAg in HBeAg-negative CHB patients receiving NAs. Our secondary aim was to identify baseline and on-treatment variables to identify low qHBsAg levels (<120 IU/mL) as a good predictor of HBsAg loss[13].

## Materials and methods

### Serum samples and study population

This is a retrospective study evaluating cryopreserved serum samples of HBeAg-negative CHB patients, receiving NAs for a minimum of 12 months, from February 1999 to October 2015. All the serum samples had been extracted in fasting conditions and centrifuged at 3000 rpm before preservation at -30°C. The serum samples were part of the private collection (C.0000956) of the IMIM (Hospital del Mar Medical Research Institute) and were identified with a number. All the data were collected and tabulated in a database with an access code to ensure patient confidentiality. Patients enrolled from 1999 to May 2006 gave verbal informed consent for the use of serum samples in biomedical research, and this consent was registered in clinical history and electronic medical records. Those enrolled from May 2006 to October 2015 provided written informed consent. The study protocol was approved by the Ethical Committee of our institution “Comitè Ètic d’Investigació Clínica—Parc de Salut Mar”, study reference 2014/5779/I, in accordance with the ethical guidelines of the 1975 Declaration of Helsinki.

All patients included were adult (>18 years old), had a baseline liver biopsy or had clinical signs of portal hypertension before antiviral treatment. We excluded patients with co-infection (human immunodeficiency virus, hepatitis C or hepatitis D), those with hepatocellular carcinoma, patients who had previously received interferon or Peg-IFN, inactive carriers, patients receiving treatment as a prophylaxis of reactivation, patients who voluntarily stopped NAs during follow-up and those with a baseline liver biopsies of less than 15 mm (length) and/or less than 6 portal triads.

### Baseline characteristics and HBsAg quantification

Demographic data of the patients, liver function tests and fibrosis stage, DNA levels, and serological status (HBsAg, HBeAg and antibodies) were retrospectively collected using the clinical history and electronic medical records.

Liver fibrosis stage was evaluated by liver biopsy before antiviral treatment. Liver biopsy was percutaneously performed using a 16-gauge Tru-Cut needle and guided by abdominal ultrasound. Samples were processed at the Pathology Department and stained with hematoxylin-eosin and Masson's trichrome. Fibrosis was staged according to the METAVIR classification[18](F0 = no fibrosis; F1 = portal fibrosis without septa; F2 = portal fibrosis with few septa; F3 = portal fibrosis with many septa and F4 = cirrhosis). Those patients with clinical signs of portal hypertension were considered as cirrhotic patients.

Antiviral treatment with NAs was based on clinical guidelines and on the physician’s decision [19–22]. Patients included in the study had been receiving NAs for more than 1 year, and NAs therapy was stopped in patients with confirmed HBsAg loss.

The HBsAg was quantified in frozen serum samples collected before antiviral treatment (baseline) and at years 1, 3, 5, 8 and over. Samples were tested and quantified for HBsAg by Electro-chemiluminescence immunoassay Elecsys® HBsAgII (Roche Diagnostic, Rotkreuz, Switzerland) according to the manufacturer’s instructions. The assay ranged from 0.05 to 52000 IU/mL but in highly concentrated samples above the upper limit a further manual dilution step was necessary to achieve results within the measuring range and multiply by the dilution factor later.

Genotype of HBV was performed by INNO-LIPA® HBV Genotyping/28708v1 (Fujirebio Diagnostics, Goteborg, Sweden) in basal cryopreserved serum samples. The lower limit of detection, according to the manufacturer’s instructions was <500 IU/mL. In those samples in which genotype could not be obtained at the first assessment, the procedure was repeated.

### Statistical analysis

Quantitative variables were expressed as medians and ranges. Categorical variables were expressed as proportions. Continuous variables were compared by the Mann–Whitney *U* test. Categorical variables were compared by the Pearson chi-square test or the Fisher exact test. Differences between patients who achieved low levels of HBsAg (<120 IU/mL) and those who did not (>120 IU/mL), were analyzed by univariate analysis. Variables showing a P value <0.05 were included in a multivariate forward stepwise logistic regression analysis to determine independent predictors of low levels of qHBsAg and seroclearance. The diagnostic accuracy of qHBsAg (log IU/mL) at each time point to identify patients reaching low levels of HBsAg was assessed using the area under the receiver operator characteristic(AUROC) curve and the 95% confidence interval (95%CI). The optimal cut-off values were selected on the basis of sensitivity (S), specificity (Sp), positive predictive value (PPV), and negative predictive value (NPV) to identify low levels of qHBsAg (<120 IU/mL) and HBsAg loss. We estimated the linear slope of qHBsAg for each group of patients (HBsAg <120 IU/mL vs. HBsAg >120 IU/mL) using a longitudinal mixed model (LMM) for repeated measurements. Cumulative incidences of low qHBsAg and seroclearance were analysed by the Kaplan-Meier method with a log-rank test. All statistical tests were two-sided and a *P* value<0.05 was considered statistically significant. The statistical analyses were performed with the SPSS® 20.0 (SPSS Inc., Chicago, IL, USA).

## Results

### Baseline characteristics of the patients

The qHBsAg (IU/mL) was evaluated in 485 frozen serum samples of 128 HBeAg-negative CHB patients collected from February 1999 to October 2015. Thirty-three patients did not fulfill the inclusion criteria and were excluded from the study: inactive carriers (n = 5), poor quality liver biopsy or basal serum not available (n = 4), treatment with interferon or pegIFN (n = 17), duration of treatment shorter than 1 year (n = 3) and indication as prophylaxis or acute reactivation (n = 4). Thus, 358 frozen serum samples of 95 HBeAg-negative CHB patients were considered for the analysis. Table 1 shows the baseline characteristics of the patients included; 73% were males, with a median age of 43; 55% were Caucasian (born in Spain or other European countries), 26% from South-East Asia and 9% from sub-Saharan Africa. Differences between European (n = 52) and Non-European (n = 43) patients are shown in Table 1. European patients were older (age: 48 vs. 37, p = 0.008), with a lower proportion of men (58% vs. 91%, p<0.001) and longer treatment duration (years: 7.1 vs. 5.2, p<0.001). However, there were no differences in fibrosis stage, alanine aminotransferase (ALT), HBV-DNA or qHBsAg levels and HBV-genotype.

**Table 1:** Baseline characteristics of the patients included in the study.

	Total cohort	Patients from Europe	Non-European	P
** **	N = 95	n = 52	n = 43	** **
**Baseline**				
Age, years	43 (18–77)	47 (19–77)	37 (18–65)	0.008
Males, n (%)	69 (73)	30 (58)	39 (91)	<0.001
**Fibrosis**				
F0-F1	70 (74)	37 (71)	33 (77)	0.5
F≥2	25 (26)	15 (29)	10 (23)	
**ALT, IU/mL**	39 (11–252)	40 (11–213)	37 (13–252)	0.9
DNA, IU/mL	23101 (10–1.68·10^8^)	19148 (10–1·10^8^)	28450 (49–1.68·10^8^)	0.2
<20000	45 (47)	28 (54)	17 (40)	0.2
>20000	50 (53)	24 (46)	26 (60)	
qHBsAg, IU/mL	4108 (2.61–187330)	3701 (2.6–187330)	5493 (234–33702)	0.3
<1000	14 (15)	9 (17)	5 (12)	0.4
>1000	81 (85)	43 (83)	38 (88)	
Genotype, n(%)*				
A	14 (19)	8 (21)	6 (17)	0.7
D	39 (54)	22 (58)	17 (49)	
Others (B, C, E, F, G)	20 (27)	8 (21)	12 (34)	
IL28, n(%)**				
CC	35 (44)	16 (36)	19 (54)	0.01
CT	37 (46)	27 (60)	10 (29)	
TT	8 (10)	2 (4)	6 (17)	
**Antiviral treatment**				
ETV/TDF	72 (76)	33 (64)	39 (91)	0.002
Others (LAM,ADV,LdT)	23 (24)	19 (36)	4 (9)	
**Treatment initiation**				
<2006	13 (14)	12 (23)	1 (2)	0.003
>2006	82 (86)	40 (77)	42 (98)	
**Follow-up**				
Time of treatment, years	5.95 (1–15)	7.1 (2–15)	5.2 (1–12.4)	<0.001
< 6 years, n (%)	49 (52)	20 (38)	29 (67)	0.005
> 6 years, n (%)	46 (48)	32 (62)	14 (33)	
HBsAg<120 IU/mL, n (%)	13 (14)	12 (23)	1 (2)	0.003
HBsAg seroclearance, n (%)	4 (4)	4 (7)	0 (0)	0.06

Quantitative variables shown as median and ranges, ALT alanine aminotransferase, LAM lamivudine, ADV adefovir, TDF tenofovir, ETV entecavir, LdT telbivudine

*Data available of 73 patients

**Data available of 80 patients

The first antiviral treatment included TDF in 44% of patients, ETV in 32%, LAM in 9%, adefovir (ADV) in 9% and telbivudine (LdT) in 5%. Among 9 patients receiving LAM, 7 switched to ADV and later to TDF and 1 to ETV during follow-up. Among 9 patients treated with ADV, 8 switched to TDF and 1 to ETV. The 5 patients receiving LdT switched to TDF. Only one patient with TDF switched to ETV due to renal failure.

### Predictors of low qHBsAg levels and seroclearance

The aim of our study was to identify baseline and on-treatment predictive variables to achieve low levels of qHBsAg (<120 IU/mL) as a good predictor of HBsAg loss in HBeAg-negative CHB patients receiving NA as reported by Chen et al.[13]. Thirteen patients out of 95 (13.68%) achieved qHBsAg <120 IU/mL during follow-up. The median level of qHBsAg at baseline was 4108 IU/mL (3.6 log IU/mL). On comparing patients with qHBsAg >1000 IU/mL (n = 81) to those with qHBsAg <1000 IU/mL (n = 14) at baseline, there were no differences regarding age, gender, fibrosis stage, HBV DNA, HBV-genotype, ALT levels or HBsAg seroclearance (p>0.05 in all cases)(data not shown).

Comparison of patients with low qHBsAg or those who achieved a reduction during follow-up (n = 13) with patients not achieving a reduction (n = 82) showed that 92% vs. 49% were Europeans (p = 0.003), had received NAs for a longer period of time (8 vs. 5.9 years; p = 0.001) of more than 6 years (77% vs. 44%; p = 0.003) with LAM (31% vs. 6%, p<0.009) or ADV (23% vs 7%, p = 0.009)(Table 2). The rate of patients with baseline qHBsAg below 1000 IU/mL was higher in those achieving low levels (<120 IU/mL)(39% vs. 11%; p = 0.02). However, among 5 patients with qHBsAg <1000 IU/mL, 3 already showed low qHBsAg (<120 IU/mL) at baseline, limiting its use as a predictive variable. Thus, these 3 patients (two over 62 years of age, with advanced fibrosis and receiving NAs during 5.3 years) were excluded from the kinetics analysis. After excluding these 3 patients, there were no differences between those with qHBsAg lower or higher than 1000 IU/mL to predict low levels during follow-up (20% vs. 11%; p = 0.3). On multivariate analysis, only the median treatment duration was longer in patients achieving low qHBsAg (8 years) compared to those who did not (5.9 years)(OR 1.2; CI 95% 1–1.45; p = 0.04)(Table 2).

**Table 2:** Characteristics of the patients included according to qHBsAg during follow-up.

	qHBsAg <120 IU/mL	qHBsAg >120 IU/mL	P
			
**Baseline**	N = 13*	**N = 82**	
Age, years	52 (19–77)	41 (18–66)	0.33
Males, n (%)	9 (69)	63 (73)	0.8
**Fibrosis (Metavir)**			
F0-F1	8 (62)	63 (73)	0.3
F≥2	5 (39)	23 (27)	
**ALT, IU/mL**	33 (17–177)	40 (11–252)	0.9
DNA, IU/mL	2864 (10–1·10^8^)	32998 (49–1.68·10^8^)	0.13
<20000	9 (69)	36 (44)	0.09
>20000	4 (31)	46 (56)	
qHBsAg, IU/mL	1822 (137–187330)	4863 (245–54924)	0.14
<1000	5 (39)	9 (11)	
>1000	8 (61)	73 (89)	0.02
Genotype, n(%)**			
A	1 (17)	13 (19)	0.87
D	2 (33)	37 (55)	
Others (B,C,E,F,G)	3 (50)	17 (26)	
IL28, n(%)***			
CC	5 (56)	30 (42)	0.7
CT/TT	4 (34)	41 (58)	
**Origin, n(%)**			
European	12 (92)	40 (49)	0.003
Non-European	1 (8)	42 (51)	
**Treatment initiation**			
<2006	5 (38)	8 (10)	0.05
>2006	8 (62)	74 (90)	
**Antiviral treatment**			
**ETV/TDF**	5 (38)	67 (82)	0.001
**Others (LAM,ADV,LdT)**	8(62)	15 (18)	
**Follow-up**	N = 13*	**N = 82**	
**Length of treatment, years**	8(2–15)	5.9 (1–14)	0.001
< 6 years, n(%)	3 (23)	46 (56)	0.003
> 6 years, n(%)	10 (77)	36 (44)	
**Delta (Δ) of HBsAg (Log IU/mL)**	N = 10*	N = 82	
1 year	0.12	0.03	0.008
3 years	0.45	0.12	<0.001
5 years	0.82	0.21	<0.001
8 years	1.2	0.29	0.001
10 years	1.8	0.59	0.009
**HBsAg seroclearance, n (%)**	4 (40)	0 (0)	<0.001

Quantitative variables shown as median and ranges, ALT alanine aminotransferase, LAM lamivudine, ADV adefovir, TDF tenofovir, ETV entecavir, LdT telbivudine. Thirteen out of 95 patients (13.68%) achieved low levels of qHBsAg (< 120 IU/mL). However, 3 patients had low levels of qHBsAg (<120 IU/mL) at baseline and were excluded from the kinetic analyses of qHBsAg(*).

** Data available of 73 patients

***Data available of 80 patients

During follow-up, 4 patients (4.21%) achieved HBsAg clearance. The median time to HBsAg clearance was 6.04 years (range: 1.7–8.1). All patients with HBsAg clearance were European (p<0.05), 3 were women (p<0.05), 2 had received ADV, and 2 TDF. The NAs therapy was withdrawn in all patients that achieve HBsAg seroclearance and HBsAg did not re-appear during the follow-up.

There were no differences in age, fibrosis stage, HBV DNA, ALT and qHBsAg at baseline (data not shown). We could only determine HBV-genotype in 2 of these 4 patients, one was genotype A and the other was a genotype G. The patients presenting HBsAg clearance showed a greater qHBsAg (log IU/mL) reduction (median), compared to those who did not show it at years 1 (0.34 vs. 0.03, p = 0.047) and 3 (1.12 vs. 0.1, p<0.001). In all the patients with HBsAg clearance the decline in qHBsAg at year 3was >0.3 log IU/mL. In contrast, none of the patients with a reduction of HBsAg>0.3 log IU/mL after 5 years of NAs therapy achieved HBsAg seroclearance.

### Kinetics of HBsAg levels and regression model during NAs therapy

**Fig 1:**
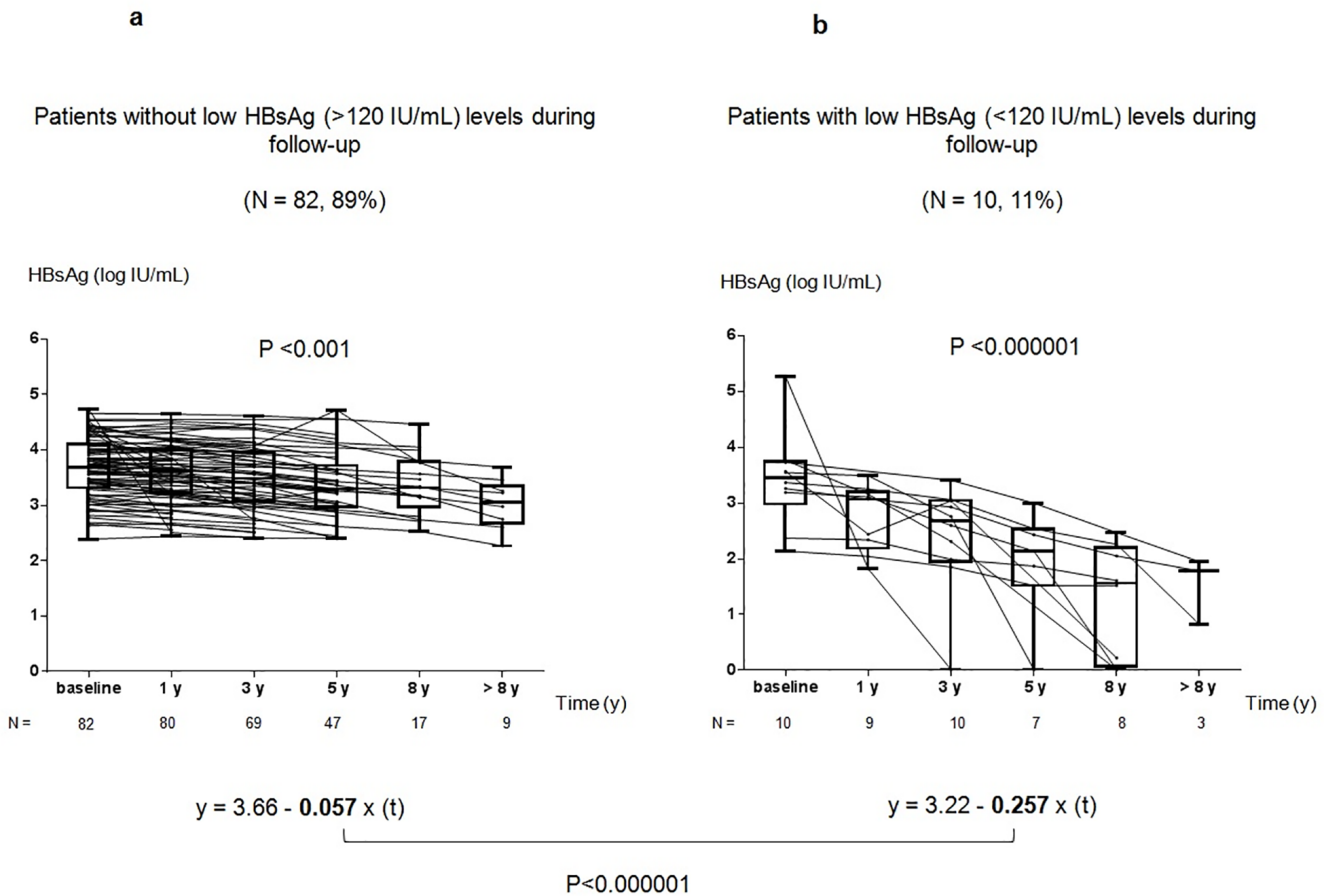
**A and B. Different kinetics of qHBsAg in HBeAg-negative CHB patients receiving NAs.** Fig 1A shows the kinetics of qHBsAg (log IU/mL) in patients who did not achieve low levels (>120 IU/mL) during follow-up (n = 82). Fig 1B shows the kinetics of qHBsAg in patients who achieved low levels (<120 IU/mL) during follow-up (n = 10). Using a mathematical mixed model for repeated measurements, the slope in patients who achieved low levels of HBsAg (y = 3.22–**0.257**x(t)) was significantly greater compared to those who did not (y = 3.66–**0.057**x(t)) (p <0.001).

Considering the lack of baseline characteristics to predict low levels of HBsAg (<120 IU/mL) we evaluated the kinetics of qHBsAg during follow-up. The reduction of qHBsAg (log IU/mL) at years 1 (n = 92), 3 (n = 81), 5 (n = 55), and 8 (n = 23) was 0.04, 0.13, 0.28, and 0.6 (p<0.001), respectively. However, patients showed two different kinetics of qHBsAg (Fig 1A and 1B). The median qHBsAg (log IU/mL) of patients achieving low levels (<120 IU/mL)(n = 10) versus those who did not (n = 82) were different after the first year: baseline (3.42 vs. 3.69; p = ns), year 1 (2.75 vs. 3.59; p<0.001), year 3 (2.39 vs. 3.52; p<0.001), year 5 (1.93 vs. 3.40; p<0.001), and year 8 (1.27 vs. 3.36; p<0.001).

In order to demonstrate the presence of two different kinetics of qHBsAg decline we used the LMM for repeated measurements of HBsAg levels (log IU/mL). The decline of qHBsAg (log IU/mL x year) during therapy was -0.102 and the median time to achieve HBsAg loss was 6.04 years. However, the slope was significantly greater in patients who achieved low HBsAg levels during follow-up (-0.257) compared to those who did not (-0.057) (p<0.001). The median levels of qHBsAg and the complete mathematical mixed model are shown in Fig 1A and 1B for better understanding.

### Diagnostic accuracy of qHBsAg decline to predict low HBsAg levels and seroclearance

**Fig 2:**
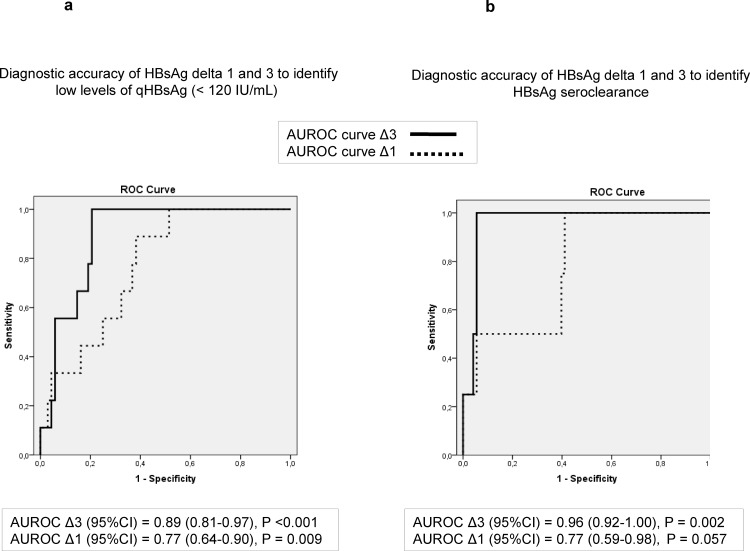
**A and B. Diagnostic accuracy of qHBsAg delta at 1 and 3 years to identify low levels of HBsAg (Fig 2A) and seroclearance (Fig 2B) during follow-up.** AUROC curve of delta at year 1 (Δ1) is depicted as a dotted line and delta at year 3 (Δ3) as a solid line.

The speed of qHBsAg decline was evaluated as the differences of qHBsAg values (log IU/mL) from baseline to year 1 (Delta1, Δ1), 3 (Delta3, Δ3) and 5 (Delta5, Δ5). Patients achieving low HBsAg levels (<120 IU/mL) during follow-up showed higher Δ1 (0.12), Δ3 (0.45) and Δ5 (0.82) values compared to those who did not achieve them (Δ1 of 0.03, Δ3 of 0.12 and Δ5 of 0.21) (p<0.05 in all cases). The diagnostic accuracy (AUROC, 95%CI) to identify patients who achieved qHBsAg<120 IU/mL was 0.77 (0.64–0.9) for Δ1 and 0.89 (0.81–0.97) for Δ3 (Fig 2A). A cut-off of Δ3>0.3 log IU/mL had a S of 100%, Sp of 81%, PPV of 42% and NPV of 100% to identify patients with HBsAg <120 IU/mL during follow-up. We analyzed the baseline variables to predict the faster qHBsAg decline at third year (Δ3>0.3 log IU/mL) and only the median HBV DNA level was higher in those patients who achieved faster reduction (313372 IU/mL) compared to those who did not (16799 IU/mL)(p = 0.03)(data not shown). The diagnostic accuracy (AUROC, 95%CI) of qHBsAg decline from baseline (delta) to predict HBsAg seroclearance was 0.79 (0.6–0.98) at year 1 (Δ1) and 0.96 (0.92–1) at year 3 (Δ3)(Fig 2B). A cut-off of qHBsAg Δ3 >0.3 log IUmL-1 showed a S and NPV of 100% (in both) to predict HBsAg loss, and a Sp of 74% and a PPV of 17%.

### Probability of low HBsAg levels and seroclearance according to qHBsAg decline

**Fig 3:**
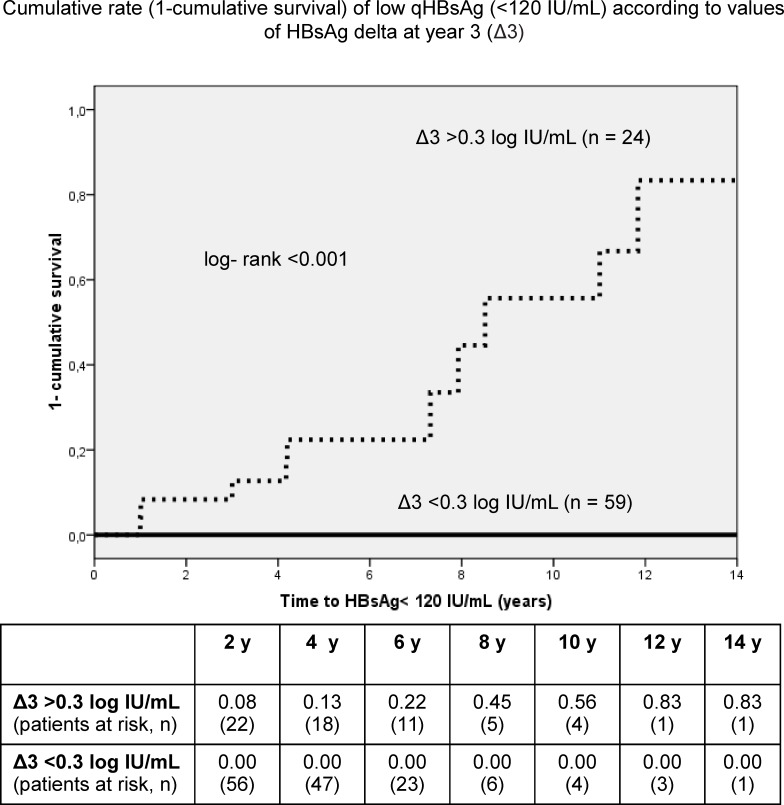
Cumulative probability (1-cumulative survival) of low qHBsAg according to values of HBsAg delta at the third year. The cumulative probability of patients with delta at year 3 (Δ3) >0.3 log IU/mL (n = 24) is depicted as a dotted line and those with Δ3 <0.3 log IU/mL (n = 59) as a solid line.

**Fig 4:**
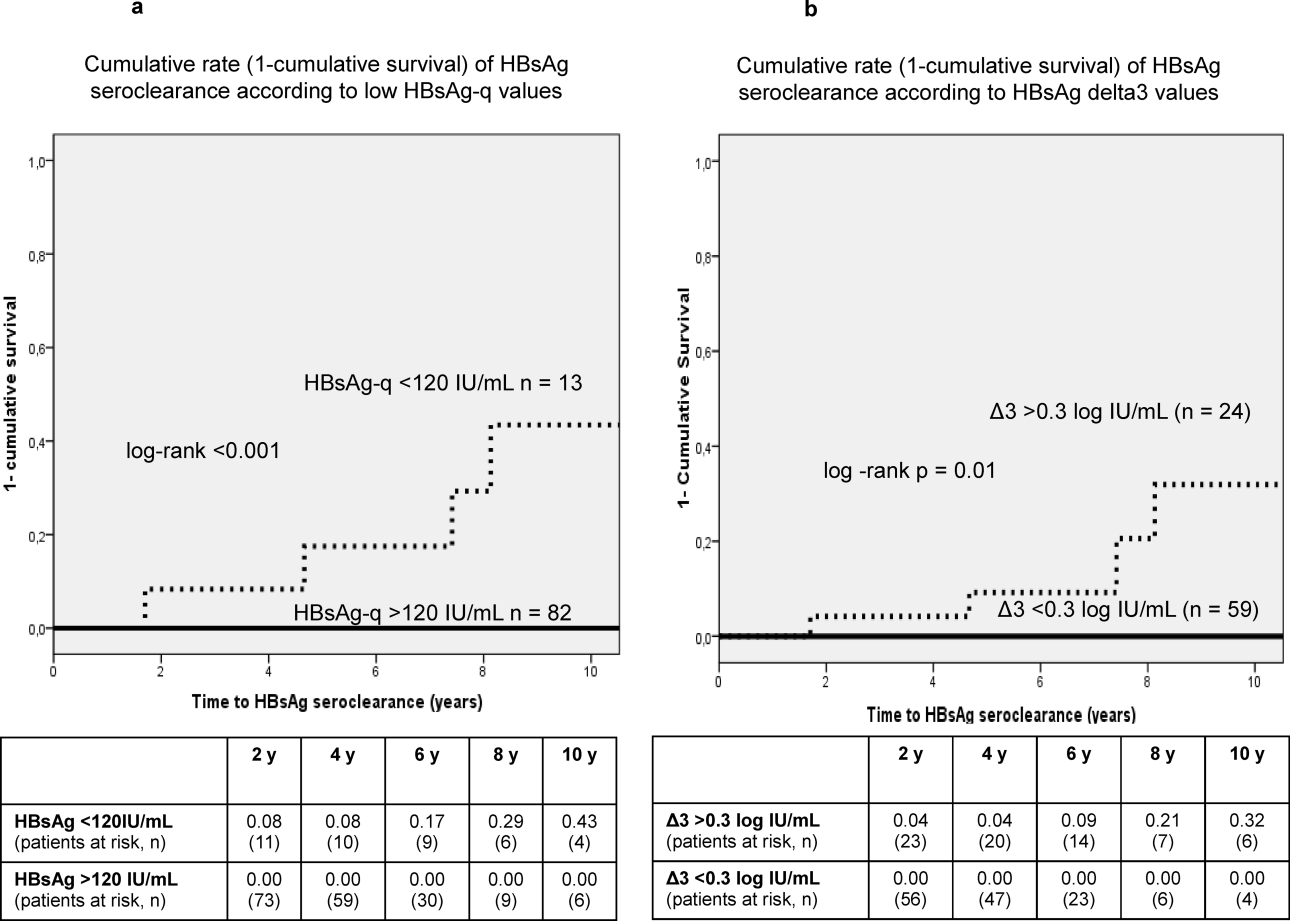
**A and B**. **Cumulative rate (1-cumulative survival) of HBsAg seroclearance according to low levels of qHBsAg or delta at year 3.**Fig 4A. The cumulative probability of patients with low qHBsAg (<120 IU/mL) is depicted as a dotted line and those with high levels (>120 IU/mL) as a solid line. Fig 4B. The cumulative probability of patients with delta at year 3 (Δ3) >0.3 (log IU/mL) is depicted as a dotted line and those with Δ3 <0.3 (log IU/mL) as a solid line.

The cumulative probability (1-cumulative survival) to achieve low qHBsAg (<120 IU/mL) during follow-up in patients with a Δ3 >0.3 log/IUmL (n = 24) after 5, 8 and 10 years of NAs therapy was 22%, 45% and 56%, respectively (log-rank<0.001) (Fig 3). Moreover, the cumulative rate (1-cumulative survival) of HBsAg loss in patients who had achieved low qHBsAg (n = 13) was 17%, 29% and 43% at 5, 8 and 10 years, respectively (log-rank<0.001) (Fig 4A).

The cumulative rate (1-cumulative survival) of HBsAg seroclearance in patients achieving a Δ3>0.3 log IU/mL was 9%, 21% and 32% after 5, 8 and 10 years of antiviral treatment with NAs, respectively, according to the Kaplan-Meier analyses (log-rank = 0.01) (Fig 4B).

## Discussion

This study was a long-term retrospective follow-up analysis evaluating the kinetics of HBsAg levels in frozen serum samples of HBeAg-negative CHB patients receiving NAs in clinical practice. The study clearly showed, a very slow decrease of HBsAg levels (-0.1log IU/mL x year) in most patients, similar to previous reports[10, 14, 23].Thus, HBsAg seroclearance occurred in only 4% of our patients after a median time of 6 years of NAs therapy. However, it is important to note that in around 14% of the HBeAg-negative CHB patients receiving NAs, low levels or a faster decline of qHBsAg occurred, showing a greater probability to achieve seroclearance.

One of the strengths of our study is that it is focused on HBeAg-negative patients who represent the vast majority of cases in many areas, including Europe[22]. Moreover, the study describes the kinetics of HBsAg levels over a long period of treatment with NAs, with a median time of 6 years and a maximum of 15 years. Regarding the origin of our patients, 55% were Europeans. Non-European patients were younger, with a higher proportion of men and received a shorter duration of treatment which is probably explained by recent migratory movements. However, there were no differences in fibrosis stage, baseline ALT, HBV-DNA, HBV-genotype and qHBsAg levels. Genotype D was the most frequent in both groups.

Functional cure of HBV, with HBsAg seroclearance remains the therapeutic endpoint in the treatment of CHB. However, our study has demonstrated a lack of baseline characteristics to predict low levels of HBsAg (<120 IU/mL) or HBsAg loss during follow-up. Previous studies have proposed that the kinetics of HBsAg during peg-IFN therapy is more accurate to predict HBsAg seroclearance than baseline qHBsAg, HBV-DNA or ALT levels in both HBeAg-positive[7] and-negative[6]patients. However, the utility of qHBsAg during NAs therapy is less clear. Seto et al.[12] found lower median baseline HBsAg levels in patients achieving HBsAg seroclearance. In contrast, our study found no differences in baseline characteristics to predict HBsAg loss, and only a greater HBsAg reduction during the first years of antiviral treatment identified patients who would achieve lower HBsAg levels and seroclearance during follow-up. It is important to note that the study of Seto et al.[12] included 61% of HBeAg-positive patients.

It has been proposed that the slow decline of HBsAg levels in HBeAg-negative patients receiving NAs might be explained by its association with the cccDNA and the independence of viral replication[14]. A previous study suggested that a qHBsAg decline at 2 years was predictive of HBsAg loss[24]. Similarly, we observed an annual reduction of qHBsAg of -0.102log IU/mL. This reduction was identical to that described by Seto et al.[12], despite the inclusion of only HBeAg-negative patients in our study. Interestingly, we found that 3 patients had low levels of qHBsAg (<120 IU/mL) at baseline. Two of these patients had advanced fibrosis and were 63 and 77 years old, and none of the 3 cleared HBsAg during follow-up. These results are consistent with those of Jang et al. who reported lower qHBsAg in elderly patients and in those with more advanced disease[25]. In our study, we did not find differences regarding levels of qHBsAg according to HBV-genotype. These results are similar to those recently published by Marcellin et al.[26] in which the HBV-genotype did not have impact in qHBsAg levels of patients receiving NAs therapy.

We also investigated the kinetics of qHBsAg during NAs therapy and found low levels or a faster qHBsAg decline during the first 3 years of NAs therapy in 14% of HBeAg-negative patients. We analyzed the kinetics of qHBsAg to reach low HBsAg levels (<120 IU/mL) during follow-up since this has been proposed as a good predictor of HBsAg loss in HBeAg-negative patients after discontinuation of LAM treatment [13]. In patients who achieved low HBsAg levels (<120 IU/mL) the reduction of qHBsAg (log IU/mL) was accelerated. In order to demonstrate the presence of two different kinetics of qHBsAg decline (negative slope) we used the LMM for repeated measurements to identify the slope of qHBsAg (log IU/mL x year) during therapy. This negative slope was significantly greater in patients who achieved low levels of HBsAg during follow-up (-0.26) compared to those who did not (-0.06). Moreover, a difference of qHBsAg greater than 0.3 log IU/mL from baseline to the third year (Δ3) showed good accuracy (AUROC of 0.89) with a PPV of 42% to identify patients with low HBsAg levels (<120 IU/mL) during follow-up. Thus, patients who presented a faster HBsAg decline, greater than 0.3 log IU/mL during the first 3 years, had a probability of 22% to achieve low values of HBsAg after 5 years of antiviral therapy. It is important to note that the only baseline variable related to the faster decline was the median HBV-DNA levels. However, DNA levels did not predict the HBsAg seroclearance.

Additionally, our study shows that all patients with HBsAg loss during follow-up showed a reduction of qHBsAg>0.3 log IU/mL during the first 3 years of NAs therapy. Similarly, Seto et al. showed that an on-treatment reduction of HBsAg >0.166 log IU/mL/year was the optimal cut-off to predict HBsAg seroclearance[12]. However, our results have shown that the accuracy of Δ3 (AUROC of 0.96) is better than that at year 1 (AUROC of 0.79) to identify patients with HBsAg loss, with a probability of 21% after 8 years of therapy. In contrast, none of the patients with a decline of qHBsAg lower than 0.3log IU/mL or those who reduced this level after 5 years of NAs therapy achieved HBsAg seroclearance.

To identify patients with a rapid drop of HBsAg levels during NAs therapy have clinical implications[3]. 1) The reduction of HBsAg levels during NAs therapy is a good predictor of on-treatment HBsAg seroclearance[12]. 2) Duration of NAs is the best predictive variable to identify patients with sustained virological response 12 months after NAs discontinuation[27] 3) Low levels of HBsAg before NAs discontinuation has been described as good predictive variable to identify patients with higher probability of HBsAg seroclearance[13]. 4) The newest European Clinical Practice Guidelines of Hepatitis B recommend to consider discontinuation of NAs long-term therapy in non-cirrhotic HBeAg-negative patients with virological response [28]. Thus, our study clearly shows that patients with a significant decrease of HBsAg levels during the first 3 years after NAs will achieve low levels of HBsAg during the next years being possible to evaluate the discontinuation of NAs. In contrast, those patients without decrease of HBsAg levels at year 3 after of NAs will not achieve HBsAg clearance during follow-up being necessary to maintenance therapy chronically or to evaluate new antiviral drugs to achieve HBsAg clearance[29].

Our study has some limitations. It is a retrospective study with non-homogeneous treatment duration with NAs. The majority of patients were receiving ETV or TDF (76%), but those with longer follow-up started treatment with LAM or ADV. However, it does reflect the real clinical practice in Europe and the evolution of chronic hepatitis B treatments during the last decades[22]. Another limitation is that we could not determine the HBV-genotype in all of our patients. However, we did not find differences in qHBsAg at baseline or during the follow-up in those patients with genotype determination (77%) as it has been recently reported[26].

## Conclusions

Our results confirm that qHBsAg decline is very slow, and the probability of HBsAg seroclearance in HBeAg-negative patients receiving NAs is low. Nevertheless, 14% of these patients showed low levels or a faster reduction in qHBsAg. These results suggest that monitoring qHBsAg levels at year 3 of therapy with NAs could be more useful to predict treatment response than baseline variables in HBeAg-negative patients. In patients with a qHBsAg decline lower than 0.3 log IU/mL at year 3 the probability of achieving HBsAg seroclearance after a long period of antiviral treatment with NAs is very low.

## Supporting information

S1 Table (XLSX)
